# Application of High-Performance Liquid Chromatography with Fluorescence Detection for Non-Polar Heterocyclic Aromatic Amines and Acridine Derivatives Determination in Pork Loin Roasted in a Roasting Bag

**DOI:** 10.3390/foods11213385

**Published:** 2022-10-27

**Authors:** Ewa Śnieżek, Magdalena Szumska, Agnieszka Nowak, Roksana Muzyka, Beata Janoszka

**Affiliations:** 1 Department of Chemistry, Faculty of Medical Sciences in Katowice, Medical University of Silesia, Jordana Str. 19, 41-808 Zabrze, Poland; 2 Department of Air Protection, Faculty of Energy and Environmental Engineering, Silesian University of Technology, Gliwice, ul. Konarskiego 22B, 44-100 Gliwice, Poland

**Keywords:** carbolines, harmane, azaarenes, heat treatment, roasted meat, dried fruits, carcinogens

## Abstract

Heat treatment of meat can lead to the formation of carcinogenic organic compounds. The influence of dried fruits on the formation of non-polar heterocyclic aromatic amines (carbolines) and nitrogen derivatives of polycyclic aromatic hydrocarbons (azaarenes) in roasted pork loin was elucidated. Two hundred grams of fruit per 1 kg of meat were used as stuffing. Carbolines, derivatives of pyridoimidazole and pyridoindole, and azaarenes (benzoacridines and dibenzoacridines) were determined by means of high-performance liquid chromatography with fluorescence detection. The total concentration of six δ-, γ- and α-carbolines in roasted pork loin was 1.3 ng/g. This content decreased by 64%, 58%, and 54% in pork loin stuffed with prunes, apricots, and cranberries, respectively. Concentrations of β-carbolines (harmane and norharmane) increased under the influence of added fruits. The norharmane content increased the most, from 2.2 ng/g in the control sample to 12.3 ng/g in meat prepared with cranberries. The harmane content increased from 1.0 ng/g to 3.6 ng/g in meat with prunes. The total concentration of azaarenes (two benzoacridines and dibenzo[*a,c*]acridine), which was close to 0.1 ng/g, decreased in dishes with prunes and apricots by 54% and 12%, respectively. Azaarenes were not found in samples of meat stuffed with cranberries.

## 1. Introduction

The thermal processing of meat may lead to the formation of mutagenic and carcinogenic organic compounds such as heterocyclic aromatic amines (HAAs), polycyclic aromatic hydrocarbons (PAHs), and their nitrogen heterocyclic derivatives (azaarenes) [1,2,3,4]. The amount and type of these compounds in food depend on temperature, time and method of thermal processing, the kind of meat and its composition, and also on the additives and fat used for frying [1,2,5,6,7,8,9].

Considering the compounds harmful to human health present in heat-treated meat and their frequent consumption, processed red meat was classified by the International Agency for Research on Cancer (IARC) as belonging to the first group of carcinogens, i.e., direct carcinogenic agents for humans [10].

Heterocyclic aromatic amines are organic nitrogen compounds [5,11,12]. They are categorized into polar and non-polar groups. Polar HAAs are formed at temperatures ranging from 100 °C to 250 °C, in accordance with the Maillard reaction mechanism, from reducing sugars, α-amino acids and creatine [6,13,14].

Non-polar HAAs are formed at higher temperatures, mainly as a result of the thermal decomposition of amino acids and proteins. The reactive fragments and radicals formed during these processes have the potential to condense and form heterocyclic structures [5,15]. However, the degradation of amino acids begins at temperatures below 250 °C [16], and possibly for this reason, non-polar HAAs have been found in highly proteinaceous foods prepared at lower temperatures [17,18,19]. The non-polar HAAs include, among others, derivatives of pyridoimidazole (δ-carbolines) and pyridoindole (α-, β- and γ-carbolines) [5,6]. They are commonly referred to as “carbolines”. The δ-carbolines are formed mainly from glutamic acid, and the other carbolines are formed from tryptophan [6,14,20].

The International Agency for Research on Cancer assigned one of the HAAs to group 2A (probably carcinogenic to humans) and nine to group 2B, corresponding to compounds that are possibly carcinogenic to humans [21]. It is worth noting that six of these compounds belong to non-polar HAAs. These are α-, δ- and γ-carbolines. These six carbolines were selected for analysis in this work, and their chemical names are listed in Table 1. Additionally, the studies covered two β-carbolines, harmane and norharmane, which do not have a free amino group and because of that they are not directly mutagenic. These compounds belong to co-mutagens, as they can enhance the mutagenicity of other carbolines and aromatic amines [11,19]. Norharmane and harmane were determined not only in cooked meat and fish, but also in processed foods of plant origin [19,22,23,24,25,26,27]. Consumption of food containing harmane and norharmane, even at high levels, seems to be safe for healthy people [23,28,29].

Polycyclic aromatic hydrocarbons formed during food processing belong to the group of compounds very harmful for human health [2,4]. The permissible content of four PAHs in food, including the carcinogen benzo(a)pyrene (BaP), is set by the European Commission Regulation. For heat-treated meat and meat products (grilled and barbecued), it is 1 ng/g of BaP [30]. In many thermal processes, along with PAHs, heteroaromatic nitrogen derivatives—azaarenes—may be formed. Studies show that azaarenes, particularly benzoacridine and dibenzoacridine isomers and their methyl derivatives, are cytotoxic, genotoxic, teratogenic, and muta- and carcinogenic [31,32,33]. 

Azaarenes may be formed in food similarly to carbolines—as a result of the thermal decomposition of organic material containing nitrogen, mainly from aromatic amino acids, particularly from tryptophan [34]. Model tests with the use of α-amino acids showed that the number and kind of azaarenes being formed depend on the temperature and composition of the mixture of thermally decomposed compounds. It is assumed that Maillard reaction products may also be thermally decomposed to acridine derivatives [3,35,36]. So far, not many works on azaarenes in food have been published [3,36,37]. These studies involved the determination of acridine derivatives in grilled and smoked meat products. Small amounts (ten times lower than the concentration of HAAs) were also found in pan-fried pork samples [38].

Better knowledge about the presence of carcinogenic compounds in meat products and their harmful activity leads to the search for recipes that can limit the formation of harmful compounds. Various studies indicate that food additives containing antioxidants can reduce HAA formation [2,12,20,39,40,41]. Such additives include, for example, spices (onion, garlic, paprika, black, Sichuan and chili pepper, herbs), fruit extracts, and also marinates with herbs, wine or beer, tea, and olive oil [7,42,43,44,45,46,47]. 

Antioxidants can inhibit various Maillard reaction pathways and prevent the formation of HAAs by quenching and scavenging free radicals [48,49]. Studies also showed that phenolic compounds are able to scavenge reactive carbonyls (needed for HAA formation) formed during proteinaceous food thermal treatment, even under common cooking conditions [12,13,39]. However, some natural additives can increase HAA concentrations [13,46,47,48,49,50]. Some phenolics converted into quinones may act as reactive carbonyls, and they can promote HAA formation [13]. There were significant differences in the additives’ effects on HAA content for β-carbolines, whose concentration increased in spiced meat dishes while the content of other HAAs decreased [6,49,50]. In other studies, it was demonstrated that natural ingredients reduced the amount of all HAAs, including harmane and norharmane, in a range depending on the cooking method [42,51,52,53].

In the literature, there is not much information on natural additives’ influence on azaarenes’ formation in food [38]. Nonetheless, some studies indicate a positive relationship between the reduction in PAH concentrations and the antioxidant potential of additives [41,54]. Antioxidants can deactivate free radicals, which are intermediates of PAHs formed at high temperatures [41,54,55]. It is likely that plant additives can also reduce the concentration of PAH nitrogen derivatives in heat-treated meat.

Although the concentrations of HAAs and azaarenes in food products are low (μg/kg), frequent exposure to such compounds may be harmful to human health. This is why science has tried to find simple ways to cook meat dishes that reduce the amount of cancer-causing compounds that people are exposed to [12,17,20,41]. The aim of this study was to investigate the effect of three selected dried fruits on the formation of eight carbolines (Glu-P-1, Glu-P-2, AαC, MeAαC, harmane, norharmane, Trp-P-1, Trp-P-2) and five azaarenes (two benzoacridines and three dibenzoacridines) in dishes from pork loin by oven roasting using roasting bags. This is a simple and popular cooking technique. In Poland, there is a tradition of preparing meat dishes stuffed and roasted with fresh or dried fruits. We found no information in the scientific literature on the effect of prunes, dried apricots, and cranberries on the formation of mutagenic and carcinogenic heterocyclic nitrogen compounds. 

## 2. Materials and Methods

### 2.1. Reagents and Materials

The carbolines and azaarenes analytical standards (purity above 98%) used in the study are listed in Table 1.

Two carbolines, harmane and norharmane, were bought from Sigma Aldrich (Buchs, Switzerland), and the other from Toronto Research Chemicals (North York, ON, Canada). Two azaarenes (B*a*Acr and B*c*Acr) were purchased from Aldrich, and the other standards were from Promochem (Wessel, Ge*r*many). Standard stock solutions (each 0.2 g/L) were used to make standard mixtures (1 mg/L) of HAAs in methanol and azaarenes in acetonitrile.

Water was obtained from a simplified water purification system (Millipore, Vienna, Austria). The solvents (all HPLC-grade) used for the mobile phases and for extraction (dichloromethane, methanol, acetonitrile, toluene, ammonium hydroxide), sodium hydroxide, hydrochloric acid, and ammonium acetate (all analytical-reagent grade) were bought from Avantor™ Performance Materials (Gliwice, Poland). The buffer was made using 85% phosphoric acid from Merck (Darmstadt, Germany) and triethylamine, HPLC grade (Fisher Scientific, Leicester, UK). Extraction columns with diatomaceous earth (Extrelut, 20 mL) were purchased from Merck (Darmstadt, Germany). Solid phase extraction (SPE) columns with propyl sulfonic acid (SPE-PRS, 500 mg) and octadecylsilane (SPE-C_18_, 500 mg) were bought from J.T. Baker (Avantor™ Performance Materials BV, Deventer, The Nederland). Dichloromethane (4 mL) was used to precondition SPE-PRS columns, whereas methanol (10 mL) and water (10 mL) were used to precondition SPE-C_18_ columns. Before being injected into the high-performance liquid chromatography (HPLC) system, all solutions were passed through a 0.45 µm filter (Millipore, Bedford, MA, USA).

### 2.2. Dried Fruits

Dried fruits (prunes, apricots, and cranberries) used for stuffing pork loin were bought at an amount of 5 packs of each kind, 200 g each, in a local market. The information on the prune packs, of Polish production, said that they were without preservatives. Dried apricots, of German production, were preserved with SO_2_. Dried cranberries, of German production, had sorbic acid (hexa-2,4-dienes acid) as a preservative.

Fruits of each kind were put together and crushed with a knife and a blender. This allowed for the averaged parts of each fruit type to be acquired, and 200 g of each fruit type was used to fill 1 kg of pork loin.

### 2.3. Meat Roasted in an Electrical Oven with the Use of Roasting Bags

The investigation focused on meat dishes made from pork loin that were cooked in households using recipes that are popular in Poland. For the experiment, two whole pork loins coming from a single pig were bought. They were specially ordered from a local butcher.

Before making the meat dishes, the fat and bones were removed from the meat. The meat was divided into 4 portions, weighing 1 kg each. In three of them, a cut was made with a knife on both sides of the meat portion so that an aperture of 3 cm in diameter was created deep along the loin. This hole was filled tightly with crushed dried fruit of a given type at an amount of 200 g/kg of pork. The fourth portion was left without stuffing. Each dish was 20 cm long, 10 cm wide, and about 18 cm in perimeter. Each of the dishes was separately wrapped in aluminum foil and kept at a temperature of 4 °C for 12 h. After removing the foil, each meat portion was put separately in a roasting bag (of Polish production). The meat did not touch the bag from the inside. Both ends of the bag were tightly closed with a clip attached by the producer. The portion of pork loin was put in the middle of a baking tray and put into an oven heated to 200 °C. Each dish (i.e., pork loin without additives, with prunes, apricots, or cranberries) was roasted separately for 30 min, at a temperature of 200 °C, and then the temperature was lowered to 180 °C, and the dish was roasted for another 60 min. Ten minutes before the end of roasting, the bag was cut to brown the roast.

After cooling, the dishes were cut open, and the fruit additives were carefully scraped away with a knife from the inside of the roasts. The meat from each dish was minced two times in an electric mincer. From 1000 g of raw meat, 520 g of pork roast (prepared without additives), 552 g when prepared with prunes, 570 g with dried apricot, and 572 g with dried cranberries were obtained.

### 2.4. Extraction of Carbolines and Azaarenes from Meat

The methodology of carboline isolation from meat samples was described in detail in our previous paper [56]. The multistage extraction and clean-up procedure was first used by Gross and Grüter [57]. After some modifications, it was applied by us for the isolation of polar heterocyclic amine and azaarene fractions [38], as well as carbolines [56].

The initial step of the procedure involved the homogenization and alkaline hydrolysis of meat samples. For this purpose, 90 mL of NaOH solution (1 mol/L) was added to a 30 g meat sample and mixed in a homogenizer (Med. Instruments, Warsaw, Poland) for 3 h. The hydrolysate was then divided into six 20 g portions, each containing 5 g of meat. A standard mixture containing eight carbolines and three azaarenes (B*c*Acr, DiB*ac*Acr, and DiB*ah*Acr) was added to two portions, yielding spiked samples with 10 ng/g and 40 ng/g of meat. Each of the 6 portions of the alkaline hydrolysate was separately subjected to a multi-step extraction procedure. Its course was as follows: 15 g of Extrelut was poured into 20 g of hydrolysate, and 10 mL of NaOH solution (1 mol/L) was added. The whole mixture was mixed and introduced into a polypropylene column. Nitrogen-containing compounds (HAAs and azaarenes) were eluted from Extrelut directly onto SPE-PRS columns by the means of 60 mL of a mixture containing CH_2_Cl_2_ with 5% toluene. These SPE-PRS columns were dried, washed with 6 mL of 0.01 mol/L HCl and water (2 mL), and next connected to the SPE-C_18_ column. The formed tandems (SPE-PRS with SPE-C_18_) were washed with 20 mL of 0.5 mol/L ammonium acetate solution (pH 8). After separation, each SPE column was washed with water (10 mL) and dried. From both columns, the nitrogen heterocyclic compounds were eluted with 4 mL of a mixture of 9 parts methanol to 1 part ammonia water. The extracts eluted from the SPE columns with C_18_ phase contained α-carbolines and δ-carbolines, whereas the extracts from the SPE-PRS column contained β- and γ-carbolines, as well as azaarenes.

Because of the low levels of HAAs and azaarenes in food, the appropriate extracts from four hydrolysates (without added standards) were mixed together. This yielded extracts equivalent to 20 g of cooked meat.

The procedure described above (including the steps of hydrolysis, Extrelut extraction, SPE-PRS, and SPE-C_18_) was carried out three times for meat samples of each of the four dishes tested.

The evaporated α- and δ-carboline fractions were dissolved in methanol prior to HPLC analysis. In this process, 500 µL of solvent was used for spiked samples and 200 µL was used for unspiked ones. Fractions containing β- and γ-carbolines, as well as azaarenes, were divided into two equal parts. One part was checked for carboline content, and the second part was analyzed for azaarene content using the HPLC technique. The part in which β- and γ-carbolines were determined was dissolved in methanol, and the other one, in which azaarenes were determined, was dissolved in acetonitrile, using volumes of 250 µL for spiked samples and 100 µL for unspiked samples, respectively.

### 2.5. Determination of Carbolines and Azaarenes by HPLC with Fluorescence Detection (FLD)

The HPLC Ultimate 3000 TSL analytical system from Dionex Softron (Germering, Germany) was used for the quantitative analysis of carbolines and azaarenes. It was equipped with Chromeleon software (version 6.80 SP2 Build 2284, Dionex Softron, Germering, Germany) and a thermostat autosampler (WPS-3000 TSL, Dionex Softron, Germering, Germany), a column compartment (TCC-3200, Dionex Softron, Germering, Germany), a diode array detector (DAD) (PDA 3000, Dionex Softron, Germering, Germany), and a fluorescence detector (FLD) (Shimadzu RF-2000, Kyoto, Japan).

For the separation of carbolines and azaarenes, different chromatographic columns and separation conditions were applied. All the separations were carried out at 40 °C.

The Tosoh Bioscience (TosoHaas, Stuttgart, Germany) TSK-gel ODS 80-TM column (particle size 5 µm), 250 × 4.6 mm I.D., and a mixture of 5% acetonitrile and 95% triethylamine phosphate buffer (pH 3.3) as the mobile phase were employed for the separation of carbolines. Gradient elution was utilized for the separations; the mixture mentioned above was used for 2 min at first, then linearly raised to 25% acetonitrile for 20 min, then to 55% acetonitrile for 10 min, and stayed at 55% acetonitrile for 10 min. The flow rate was 1 mL per minute.

For azaarenes, a column designed specifically for the analysis of polycyclic aromatic hydrocarbons was used, i.e., Hypersil Green PAH (particle size 5 µm), 250 × 4.6 mm I.D., with a guard column (5 µm, 10 × 4 mm) (Thermo Scientific, Waltham, MA, USA). The separations were performed under isocratic conditions, using a mixture of 85% acetonitrile and 15% water as a mobile phase (flow rate of 1.0 mL/min).

In order to obtain signals of maximum intensity on the chromatograms for the determined carbolines and azaarenes, fluorescence detection was carried out using excitation (Ex) and emission (Em) wavelength programs optimized for the determined compounds. The following Ex/Em wavelength program was used to determine the content of carbolines. For the determination of Glu-P-2 and Glu-P-1, the wavelengths used were 360/450 nm from 0 to 14 min; 300/440 nm from 14.01 to 18 min (norharmane, harmane); 263/400 nm from 18.01 to 26 min (Trp-P-2, Trp-P-1); and 335/410 nm from 26.01 to 30 min (AαC, MeAαC).

The Ex/Em wavelength program used to determine azaarenes was as follows: 279/404 nm from 0 to 11.5 min, (determination of B*c*Acr and B*a*Acr); 278/388 nm from 11.51 to 14.5 min, (DiB*ac*Acr); and 291/410 nm from 14.51 to 22 min, (DiB*aj*Acr, DiB*ah*Acr).

Qualitative analysis was performed by comparing the retention times recorded for standards with the values of appropriate components identified in the spiked and unspiked meat samples. The quantitative analysis was performed by using peak area values and a method based on an external standard.

### 2.6. Identification of Azaarenes by Gas Chromatography—Mass Spectrometry (GC-MS)

The remaining extract solutions, after determination by the HPLC-FLD technique, were subjected to GC-MS analysis to confirm the presence of azaarenes in food samples. A gas chromatograph, Trace 1310 Thermo Scientific (Milano, Italy), connected to a triple quadrupole mass spectrometer, TSQ 9000 (San Jose, CA, USA), was used. The samples were analyzed by means of split-less injection (2 min) into a TG-5MS GC capillary column (30 m × 0.25 mm; film thickness: 0.25 µm) (Thermo Scientific, Waltham, MA, USA). Conditions for the analysis of azaarenes were as follows: electron ionization (EI) 70 eV; helium flow rate of 1 mL/min; temperatures: injector 270 °C, interface 320 °C, ion source 320 °C; GC temperature program: 70 °C (2 min), 5 °C/min to 320 °C (5 min). One-microliter samples were injected using a TriPlus RSH auto sampler (Thermo Fisher Scientific, San Jose, CA, USA). The samples were analyzed in the full scan mode (mass range 50–550 Da). The mass spectra of azaarene standards are dominated by their molecular ion [M]^+^. The identification of azaarenes consisted of comparing retention times and the appropriate mass spectra of standards and compounds identified in meat samples. Identification was performed using the Chromeleon software (version 7.2.10) and mass spectral databases Mainlib and NIST (National Institute of Standards and Technology in USA).

### 2.7. Statistical Analysis

Statistical analysis was performed using the Statistica software, version 12 (StatSoft Polska Sp. z.o.o., Cracow, Poland). Basic parameters of descriptive statistics, such as the mean, standard deviation and relative standard deviation, were used to present the results of carboline and azaarene determination. Four meat dishes were investigated. Each was prepared from 1 kg of meat. A multi-step extraction procedure was carried out three times for each type of dish. Then, the samples were subjected to HPLC-FLD analysis. Each of the extracts isolated from the meat samples was subjected to 2-fold (carbolines) or 3-fold (azaarenes) analysis by HPLC-FLD technique. Thereby, the results in Tables 2–4 correspond to *n* = 6 or *n* = 9 repetitions. Comparison of the results of carboline analysis obtained for four meat dishes prepared without and with three different fruits as additives was carried out using the ANOVA test for variables with normal distribution and the Kruskal–Wallis test for variables not showing normal distribution. Statistical significance was defined as *p* < 0.05.

## 3. Results and Discussion

### 3.1. Determination of Carbolines by HPLC-FLD

**Figure 1:**
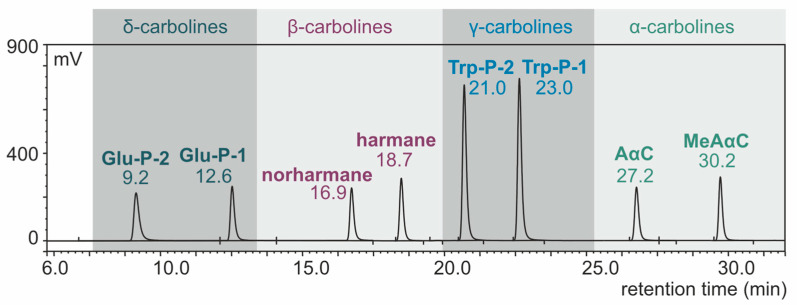
HPLC-FLD chromatogram of carboline standard mixture (concentration: 1 ng/µL, injection: 2.5 µL).

With the use of the HPLC system (conditions: TSK-gel ODS-80-TM column, buffer phosphate-triethylamine with pH 3.3, gradient elution), the components of the eight-carboline mixture were separated. The results of standard carboline mixture separation are presented in Figure 1 as a chromatogram.

With the application of the fluorescence Ex/Em program, the carbolines could be determined at a low concentration level. Based on the standard deviation (SD) of the analytical results (*n* = 6) of standard carbolines and the direction factors (b) of the corresponding calibration curves, the limits of detection (LOD) of the method were calculated using the formula LOD = 3.3 SD/b. Three times the LOD was determined to be the limits of quantitation (LOQ) of the method [58]. The LOQ expressed in ng/g of meat ranged from 0.01 (Trp-P-1) to 0.18 (norharmane) [56]. Lower (up to 2-fold) LOQ values were obtained by Xu et al., who applied the modern technique of HPLC-quadrupole-orbitrap mass spectrometry for HAA determination [59].

The least squares method was used to make calibration plots for carbolines based on the results for eight points. The plots were linear in the range of 0.1 ng to 10 ng of the compound introduced into the column for Glu-P-2, Glu-P-1, harmane, AαC and MeAαC, in the range of 0.1 ng to 5 ng for Trp-P-1 and Trp-P-2, and in the range of 0.2 to 10 ng for norharmane. The regression coefficient *r* was >0.999 for plots created for all carbolines [56].

The repeatability (intra-day precision) of the HPLC-FLD method was measured using the standard deviations (RSD) of the results obtained on the same day (*n* = 5) for samples with different concentrations of carboline (5 ng, 2.5 ng, and 0.5 ng in 10 µL of solvent), which ranged from 0.13% to 2.2%. The inter-day precision, i.e., reproducibility, carried out over 5 days, was in the range of 0.45% to 4.45%.

The recovery rates were based on the results of analyses performed for both unspiked and spiked meat samples. To calculate recovery rates, the formula [(C1-C2)/C] ×∙100% was used, where C1 is the concentration of carbolines in a meat sample with added standards (ng/g), C2 is the concentration of carbolines in a meat sample (ng/g), and C is the quantity in nanograms of the standard per 1 g of meat sample. The recovery rates ranged from 43.5% (Glu-P-1, 10 ng/g meat sample spiked) to 84.3% (harmane, 40 ng/g meat sample spiked). The recovery rates are similar to what the authors of other studies [15,50] found when they analyzed the carboline content in real meat samples.

### 3.2. Determination of Azaarenes by HPLC-FLD

**Figure 2:**
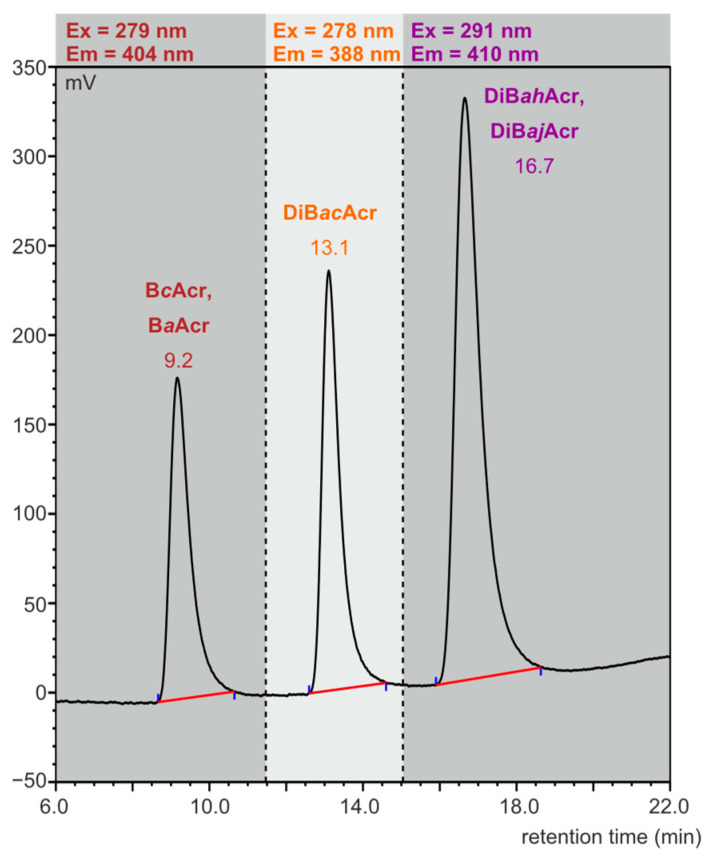
HPLC-FLD chromatogram of azaarene standard mixture (concentration: 0.1 ng/µL, injection: 2.5 µL).

Our preliminary studies showed that azaarene concentrations in meat cooked using oven bags are very low. The previously used HPLC-FLD method [38] was modified by selecting the flow rate (1 mL/min) of the mobile phase (85% acetonitrile and 15% water) in such a way that the isomers B*a*Acr and B*c*Acr, as well as the two dibenzoacridines (*ah* and *aj*), would not separate, so that at least the sum content of these compounds in the food samples could be determined. The retention times were 9.2 min for B*c*Acr and B*a*Acr, 13.1 min for DiB*ac*Acr, and 16.7 min for DiB*ah*Acr and DiB*aj*Acr. In Figure 2, a chromatogram obtained as a result of standard azaarene mixture separation is presented.

The calibration plots were created for each azaarene separately by means of the least squares method using the results obtained for six points. The plots were linear in the range from the LOQ to 1 ng of the compound introduced into the column in 10 µL of acetonitrile for all azaarenes except for DiB*ah*Acr, for which the range was from the LOQ to 0.5 ng. Regression coefficients (*r*) for the curves were above 0.999. The LOD and LOQ, as well as the repeatability, reproducibility, and recovery rate, were determined similarly to carbolines. The LOQ (ng/g) were as follows: 0.006 ng for DiB*ah*Acr and DiB*aj*Acr, and 0.01 ng for B*a*Acr, B*c*Acr and DiB*ac*Acr. The repeatability and reproducibility, which were determined using samples with different azaarene contents (i.e., 0.05 ng, 0.2 ng, and 0.5 ng in 10 µL of acetonitrile) and expressed as RSD, varied from 0.7% to 6.1% and from 3.9% to 7.2%, respectively.

In the meat samples, quantification of the total content of B*c*Acr and B*a*Acr was carried out against the calibration curve made for B*c*Acr, and the total content of DiB*aj*Acr and DiB*ah*Acr was quantified against the curve made for DiB*ah*Acr. Recovery rates of B*c*A, DiB*ac*Acr and DiB*ah*Acr ranged from 65.7% (DiB*ac*Acr, meat spiked with 10 ng/g) to 71.9% (B*c*Acr, meat spiked with 40 ng/g). These recovery rates are slightly better than those determined previously. This may be due to the less complex matrix of oven-roasted pork loin compared to pan-fried meats [38].

### 3.3. Carbolines Concentration in Meat Samples

Data from the quantitative analysis of carbolines in meat dishes prepared without and with dried fruits expressed in nanograms per gram of roasted meat are presented in Table 2 and Table 3. Carboline concentrations were in the range from “below the LOQ” (MeAαC in all meats with fruits) to 12.3 ng/g (norharmane in meat with cranberries).

Numerous factors can affect the synthesis of HAAs, so all dishes were made using pork joints that came from the same animal in order to assess the impact of particular dried fruits on the creation of non-polar HAAs. They were roasted in the same electric oven under the same temperature conditions. Salt and other spices were also not used because studies’ results show that both the composition of HAA precursors in meat and the content of water, spices, and fat used for frying can affect the formation of these compounds [1,2,24]. Since the dishes were prepared using the roasting bag method, there was no need to add oil for frying.

Carboline concentrations in pork loin samples without additives are lower than those determined by other authors and by us in dishes prepared by frying or grilling [46,47,53,56]. Meat roasted in a tightly closed roasting bag does not get very brown, despite staying in the oven for a long time. The conditions of such heat treatment are more similar to those of stewing meat. The studies by other authors also showed that the dishes prepared in an electric oven had low levels of heterocyclic aromatic amines [45,46,60].

Savas et al. compared the HAA concentrations formed in chicken meat (breast and leg) cooked in an electric oven with and without oven bags of different brands. This treatment reduced the total polar HAA content from 12 up to 69%, depending on the type of meat and bag. However, compounds from the carboline group were not detected in this study [61].

The dominant compounds in pork loin roasted without additives tested in this work are β-carbolines, norharmane and harmane. Their concentrations are 2.2 and 1.0 ng/g, respectively, and are several times higher than those of other carbolines. A probable cause is that norharmane and harmane may be formed at a lower temperature than other carbolines [6,8,19]. The precursor of these compounds in cooked meat may be tetrahydro-β-carboline-3-carboxylic acid, formed from tryptophan and aldehydes or α-keto acids present in food or formed during heat treatment. Norharmane and harmane are formed by the oxidation and decarboxylation of this acid under the influence of oxidants and free radicals present in heat-treated foods [28,62].

Literature data confirm that the β-carboline content in meat dishes is higher than the content of other carbolines. High concentrations (from a few to tens of ng/g) of norharmane and harmane were determined in fried, roasted, and grilled meats [1,9,42,52,53,63]. A β-carboline content of up to 260 ng/g was determined in pork samples [60] and marinated beef prepared according to traditional Chinese cuisine [64].

The concentrations of δ-, γ-, and α-carbolines in the samples of pork loin roasted in the roasting bags examined in our study did not exceed 0.31 ng/g (Glu-P-1, meat without additives). This value is lower than the concentrations of α-, δ-, and γ-carbolines determined in pan-fried or oven-roasted dishes made from pork, beef, and chicken, in which the content of some compounds (e.g., AαC, MeAαC, Glu-P-1) ranged from a few to tens of nanograms per gram [42,52,60,63,65]. Small amounts (0.02 to 0.14 ng/g) of Glu-P-2, Glu-P-1, AαC, MeAαC, Trp-P-1, and Trp-P-2 were determined in fried chicken and camel fillets [18]. The determined carbolines were not present in raw meat. According to literature data, HAAs are not present in such meat [5,61].

The application of stuffing with dried fruits (prunes, apricots, cranberries) at an amount of 200 g/kg of meat allowed us to obtain dishes with a lower content of all carbolines, except for norharmane and harmane, compared to that without additives.

**Figure 3:**
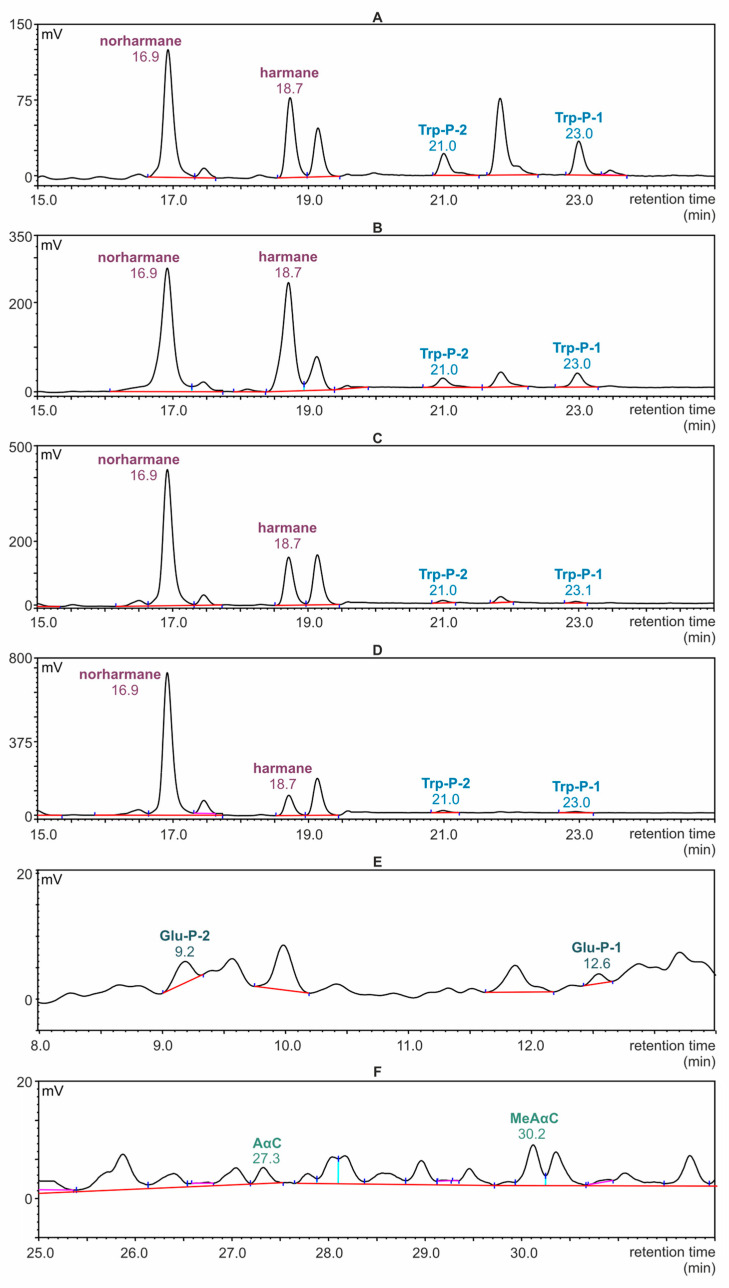
HPLC-FLD chromatograms of fractions separated from roasted meat samples. (**A**–**D**): sections of chromatograms with a selected retention time range (15–25 min) at which β- and γ- carbolines were determined. (**A**): Meat without additives; (**B**): meat with prunes; (**C**): meat with apricots; (**D**): meat with cranberries; (**E**,**F**): sections of chromatograms recorded for meat without additives; (**E**): retention time range at which δ-carbolines were determined (8–13.5 min); (**F**): retention time range at which α-carbolines were determined (25–32 min).

Selected chromatograms registered during the determination of β- and γ-carbolines in the samples of meat by the HPLC-FLD technique are presented in Figure 3A–D. The scale of the peak intensity axis in the figures corresponds to the highest peak. For this reason, in the chromatogram recorded for the meat sample with cranberries, the Trp-P-1 and Trp-P-2 signals appear to be invisible, although it was possible to integrate them for quantification. Parts of an exemplary chromatogram corresponding to α- and δ-carboline fractions isolated from the meat sample are presented in Figure 3E,F.

The total contents of the eight determined carbolines were as follows: 4.44 ng/g in pork loin roasted without additives, 10.01 ng/g in pork with prunes, 9.79 ng/g in pork with apricots, and 14.11 ng/g in pork with cranberries. These results may suggest that the use of dried fruit as meat stuffing is not beneficial. However, the increase in the total carboline content in dishes with fruit is due to an increase in the concentrations of harmane and norharmane. These compounds are not as harmful to humans as the other carbolines. Therefore, the discussion of the effects of fruit on the formation of the determined non-polar HAAs in meat dishes is divided into two parts, the first of which deals with β-carbolines, while the second part deals with δ-, γ-, and α-carbolines.

#### 3.3.1. Effect of Dried Fruits on β-Carbolines Concentration

All of the dishes prepared using pork loin stuffed with dried fruits contained norharmane and harmane at amounts significantly (*p* < 0.05) higher than those found in pork loin cooked without these additives (Table 2). The highest concentration of norharmane was determined in the samples of meat with cranberries (12.3 ng/g, i.e., 5.6 times more than in the pork loin without the additives). The highest concentration of harmane was found in the samples of meat with prunes (3.6 ng/g, i.e., 3.6 times more than in the pork loin without fruits).

In the scientific literature, few data on the effect of fruits on β-carboline formation in meat dishes are available [18,46]. Moreover, the results of the studies on the role of fruits, as well as other additives, in harmane and norharmane formation are not clear and explicit. The addition of 0.5% of pomegranate seed extract to dishes made from minced beef meat resulted in an increase in harmane and norharmane concentrations in the dishes roasted in an electric oven or fried in fat, whereas the β-carboline concentration in the dishes prepared on a charcoal grill or fried on a frying pan reduced [46]. Similarly, under the influence of 1% artichoke extract, the harmane content of pan-fried beef increased, while that of chicken prepared in the same way decreased [63]. In roasted lamb, the harmane and norharmane content increased when *Tamarix ramosissima* extract was added at a level of 0.45 g/g. Smaller amounts of this extract only reduced the content of norharmane [49].

Other studies showed that *Rosa rugose* tea extract at an amount of 0.1% added to beef meat fried in oil reduced norharmane and harmane concentrations by almost half [47]. *Chrysanthemum morifolium* flower extract added at an amount of 0.2% (*w*/*w*) to ground goat meat significantly reduced the concentration of norharmane (by 60%) in deep-fried meat and harmane (by 66%) in pan-fried meat [53]. Organic acids from tamarind, lemon, lime, and calamansi used in marinades most strongly reduced the concentration of harmane and norharmane in grilled chicken when large amounts of these additives were used, i.e., about 8–12 g/100 g of marinade [51]. Adding a marinade of freshly squeezed blueberry, raspberry, and strawberry juice (50 mL/200 g meat) to camel, beef, and chicken meat before frying reduced harmane and norharmane concentrations in all samples. The concentration of β-carbolines decreased most strongly (by 40–67%) when the marinating time lasted for 24 h [18]. In model testing with the use of chlorogenic acid, a polyphenol present in prunes and dried apricots at an amount of about 1 mg/g [66], an almost 2-fold increase in norharmane and harmane concentration was observed in relation to the control samples after the introduction of 0.05% and 0.1% of this polyphenol into minced beef meat, from which the dishes fried at 230 °C were made [67]. It seems that changes in harmane and norharmane concentrations in meat dishes under the influence of vegetable additives depend on both the type of meat and heat treatment, as well as the composition of the additives themselves.

β-carbolines as natural alkaloids were detected in some vegetables and fruits, including dried fruits [19,23,24,25,26,41,68]. Particularly high concentrations of norharmane and harmane, ranging from 16 to 644 ng/g, were determined in dark brown raisins [26]. No information in the literature about the occurrence of harmane and norharmane in prunes, apricots, and cranberries was found. In plums, however, the alkaloid tetrahydro-β-carboline (6-hydroxy-1-methyl-1,2,3,4-tetrahydro-β-carboline) was detected at a concentration of 0.11 µg/g [22]. 1,2,3,4-tetrahydro-β-carboline and 1-methyl-1,2,3,4-tetrahydro-β-carboline were determined in prunes at nanograms up to micrograms per gram levels, and their contents depended on the ripening process [69]. These compounds may be oxidized into norharmane and harmane, respectively [27]. Tetrahydro-β-carboline-3-carboxylic acid, which is a direct precursor of aromatic β-carbolines in foods due to chemical or enzyme-catalyzed oxidative decarboxylation [70], was found in apricot and plum jams and marmalades in the range of 0.1–0.2 μg/g product [71]. Recently, relatively high concentrations of carbohydrate-derived β-carbolines have also been determined in dried fruits, jams, and juices. They are formed in the reaction of tryptophan with glucose, fructose, or sucrose at temperatures above 80 °C. These β-carboline derivatives were found in higher concentrations in processed foods compared with fresh and unprocessed foods [72]. It is highly probable that the increase in harmane and norharmane concentrations found in our study in meat dishes prepared with natural additives was due to the formation of β-carbolines from ingredients present in dried fruits [25,26,27,62,70].

#### 3.3.2. Effect of Dried Fruits on δ-, γ-, and α-Carbolines Concentration

Unlike harmane and norharmane, δ-, γ-, and α-carbolines belong to the compounds which are possibly carcinogenic to humans [21]; thus, it is crucial to promote culinary procedures which can limit harmful exposure of humans to these xenobiotics.

The application of dried fruit stuffing makes it possible to obtain the roast with a relatively low content of δ-, γ-, and α-carbolines, in the range from not quantified (MeAαC in all dishes with fruits) to 0.16 ng/g (Glu-P-2, pork loin with apricots and cranberries, AαC pork loin with prunes). The decrease in the concentration in fruit-stuffed meats was statistically significant (*p* < 0.05) only for δ-carbolines (Glu-P-1, Glu-P-2) and γ-carbolines (Trp-P-1, Trp-P-2). The concentration of MeAαC in samples with added fruit did not exceed the LOQ (0.1 ng/g), and therefore was not determined. The LOQ for AαC is lower by half and amounts to 0.05 ng/g. In the case of this compound, it was possible to determine concentrations, but no statistically significant differences (*p* > 0.05) were noted for AαC content in individual dishes. A similar result, i.e., statistically similar concentrations of AαC (and also of MeAαC), was recorded in beef patties prepared with and without an extract of *Rosa rugose* tea [47].

Table 3 presents the values of the total concentration of δ-, γ-, and α-carbolines determined in samples of pork loin roasted without additives and with stuffing of prunes, apricots, and cranberries, as well as the percentage of concentration decrease determined in the dishes prepared with the addition of dried fruits.

The comparison of the total concentration of six carbolines (Glu-P-1, Glu-P-2, Trp-P-1, Trp-P-2, AαC, and MeAαC) determined in pork loin samples with fruits allows us to establish a sequence which shows how the additives can inhibit the process of δ-, γ-, and α-carboline formation. The sequence is as follows: prunes (reduction in carboline concentration by 64%) > apricots (57.8%) > cranberries (53.9%).

The dried fruits used in the study were subjected to tests [73] on the determination of antioxidant properties through the determination of ferric reducing antioxidant parameter (FRAP) and the reduction in 2,2-diphenyl-1-picrylhydrazyl radical (DPPH) by antioxidants contained in the analyzed sample. The sequence of antioxidant activity of the fruits was the same as the sequence of reduction in carboline concentration in meat under the influence of fruit, i.e., prunes > apricots > cranberries [73].

The entire amount of used dried fruit was placed inside the hole cut in the pork loin, so the meat was at all times in continuous contact, over a large surface area, with the fruit before thermal treatment and during roasting. The fruits surrounded by the meat were neither burned nor dried out. At roasting temperature, antioxidants could be released along with steam from the fruit, and these had the potential to affect the meat’s components and the formation of HAAs.

A review of the literature shows that antioxidant-rich additives such as apple juice, extracts from elderberries, grape seeds, hawthorn fruit, cherry fruit, and extract from dried apple peelings added to meat dishes at amounts of 0.1–11.5% (*w*/*w*) may inhibit the formation of compounds from the polar heterocyclic aromatic amine group by as much as 90% [44,45,74].

There are few data in the literature on the effect of fruits on the formation of non-polar HAAs (α-, δ-, and γ-carbolines) in meat dishes. Marinating camel and chicken meats even for 1 h in freshly squeezed blueberry, raspberry, and strawberry juice reduced the content of Glu-P-2, Glu-P-1, AαC, MeAαC, Trp-P-1, and Trp-P-2 in pan-fried products to 0.01 ng/g. In samples of meats marinated for 12 h, these compounds were not detected [18].

Literature data indicate that additives of plant origin with known antioxidant properties show beneficial effects for the meat consumer due to the reduction in the content of carbolines in meat dishes. The decrease in γ-carboline (Trp-P-1, Trp-P-2) and AαC concentrations was recorded under the influence of small amounts (0.1–1%) of artichoke extract (*Cynara scolymus L.*) [63], *Rosa rugose* tea extract, turmeric and lemon grass [42,45,47], *Tamarix ramosissima* extract [49], *Chrysanthemum morifolium* flower extract (0.2%, *w*/*w*) [53] and organic acids from tamarind, lemon, lime, and calamansi used as components of marinades [51].

The dried fruits selected for this study (plums, apricots, and cranberries) are rich in polyphenols such as phenolic acids, gallic acid, chlorogenic acid (3-O-caffeoylquinic acid), caffeic acid, p-coumaric acid, and flavonoids [66]. In addition, p-hydroxybenzoic acid may be present in cranberries and ferulic acid in prunes. These fruits also contain quercetin (more than 20 mg/100 g in apricots). Unlike the others, apricots also contain rutin (quercetin-3-O-rutinoside) and carotenoids, mainly β-carotene (a few to several µg/g) [66]. Rutin added to minced meat at amounts of 0.002–0.02% significantly inhibited (by nearly 100%) the formation of AαC in a roast beef system [52]. The descriptions available in the literature on the effects of polyphenolic compounds on the formation of heterocyclic amines are related mainly to polar HAAs [29,68]. Out of the antioxidants listed above, chlorogenic acid present in apple extract was responsible for reducing the concentration of imidazoquinoxaline (MeIQx) in meat [74], although it enhanced the formation of 2-amino-1-methyl-6-phenylimidazo[4,5-b]pyridine (PhIP). *Chrysanthemum morifolium* flower extract rich in quercetin glucosides and rutinosides and also in chlorogenic acid reduced the content of some HAAs in the range from 9% (AαC) to 82% (harmane) in goat meat patties, with the degree depending on cooking temperatures and methods [53]. Artichoke extract, containing mono- and dicaffeoylquinic acids and flavonoids, inhibited the formation of total HAAs by 6–98% in pan-fried and oven-roasted beef and chicken meat [63]. The Trp-P-2 concentration was also lowered by this additive [63]. Carotenoid extract from tomatoes as well as the main tomato flavonoid quercetin reduced the concentration of polar HAAs, namely imidazoquinoxalines, in a mixture of their precursors and in meat juice system [48]. Studies have also shown that phenolic compounds (chlorogenic, ferulic, caffeic acids, and rutin) mixed in the same ratios as they are present in spices may have synergistic effects on β-carbolines, but an antagonistic one on the formation of some polar HAAs [68].

The exact mechanism for the synthesis of carbolines other than harmane and norharmane was not found in the literature [15,17,62]. The precursor of α- and γ-carbolines is tryptophane, as it is for β-carbolines [6,14,20]. However, these carbolines differ from harmanes by the presence of an amino group and a different position of the nitrogen atom in the heterocyclic aromatic ring [6,20]. δ-carbolines have three heterocyclic nitrogen atoms, and their main precursor is glutamic acid [6,14,20]. Carbolines belong to the group of pyrolytic HAAs, which are formed at higher temperatures than polar heterocyclic amines [5,14]. Thermal decomposition of amino acids and other meat components may lead to the formation of free radicals [13,14,34,35,48,55]. These reactive molecules are involved in the formation of polar HAAs, β-carbolines and probably also in the generation of other carbolines. The inhibitory effect of polyphenols on HAAs formation may be due to the uptake of reactive carbonyl forms involved in the formation of HAAs, as well as the scavenging of free radicals formed during the heat treatment of meat [20,41]. It cannot be ruled out that polyphenolic compounds from the fruits may have influenced the formation of β-carbolines. However, when meat was stuffed with dried fruits, harmane and norharmane were probably formed mainly from the tetrahydro-β-carboline-3-carboxylic acid present in these additives [70,71,72], as discussed earlier.

It is also worth noting that tetrahydro-β-carbolines, which may be present in the dried fruit, also exhibit antioxidant activity and may act as free radical scavengers. The indole ring of tetrahydro-β-carbolines can form an indolyl cation or neutral radical via single electron transfer, while acting as a free radical scavenger. The indolyl radical can be further oxidized into a fully aromatic β-carboline, such as harmane and norharmane, in the case of tetrahydro-β-carboline-3-carboxylic acids, or decomposed into unknown compounds [71,75,76]. However, the expected contribution of these compounds to the fruits’ total antioxidant activity is likely to be minor due to the low concentrations of these compounds compared to vitamins, carotenoids, or phenols [71,75].

One of the main factors affecting the formation of heterocyclic aromatic amines in high-protein foods is the sugar content in the thermally processed product [14,20]. The investigations showed that even a small amount of sugar added to minced fish inhibits the formation of AαC, and MeAαC, while the addition of 19% sugar causes a decrease in the concentrations of these compounds almost by half [77]. Additionally, a decrease in δ-, γ-, and α-carboline concentrations was recorded under the influence of fructooligosaccharides added at an amount of 1 g/100 g of minced beef meat, while the addition of sugar at an amount of 5 g caused increases in norharmane, harmane, and AαC concentrations [65]. Dried fruits, namely prunes, apricots, and cranberries, contain about 12.5 g of fructose per 100 g of product [78]. The application of 200 g of fruits for 1 kg of pork loin may introduce about 2.5 g of fructose for 100 g of meat. Thus, it is very probable that the considerable decrease in α-, δ-, and γ carboline concentrations recorded in the present work was the result of the sugar concentration increase in the prepared dishes.

### 3.4. Azaarenes in Meat Samples from the Dishes Prepared with and without Vegetable Additives

The azaarene concentrations in the investigated dishes prepared from pork loin roasted in a roasting bag were lower by an order of magnitude than the carboline concentrations. The total content of B*a*Acr and B*c*Acr was only 0.07 ng/g, and that of DiB*ac*Acr was 0.03 ng/g. The concentration of DiB*ah*Acr and DiB*aj*Acr was lower than the LOQ, which is a satisfying result, because DiB*ah*Acr was classified by the IARC as belonging to group 2B (possibly carcinogenic) [32] and DiB*aj*Acr was upgraded to group 2A (probably carcinogenic) in 2013 [33].

The azaarene concentrations in the dishes with prunes and dried apricots decreased by 54% and 12%, respectively (Table 4). In the pork loin with cranberries, no azaarenes were found at all. Azaarenes may be formed as a result of radical processes during the thermal decomposition of organic matter rich in nitrogen, mainly from tryptophan [34,35]. Polyphenols present in fruits potentially affected the radical mechanism of azaarene formation, similarly to in the case of carbolines. The possibility of a concentration reduction for the compounds from the azaarene group was stated in our earlier studies on the use of onion and garlic as additives in fried pork dishes [38].

The applied thermal processing of meat, i.e., roasting the meat in a roasting bag in an electric oven, allowed us to obtain dishes with a considerably lower content of azaarenes than in fried or grilled dishes, in which the concentrations of these compounds may reach a few nanograms per gram [3,38].

**Figure 4:**
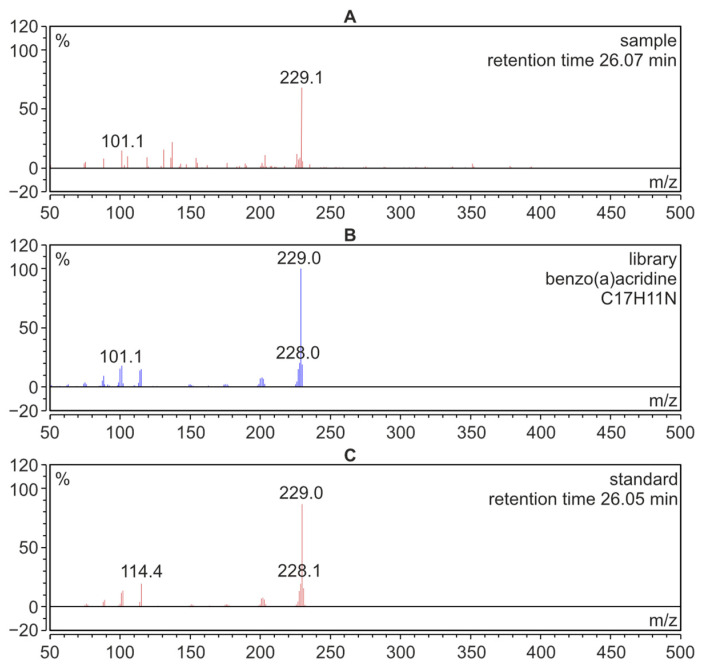
GC-MS mass spectra; (**A**): benzo[*a*]acridine identified in a sample of pork roasted without additives; (**B**): B*a*Acr from the Mainlib/NIST library resources; (**C**): spectrum of the B*a*Acr standard.

After the analysis of azaarenes performed using the HPLC-FLD technique, the residue solutions were used for qualitative determinations by means of the GC-MS technique. These investigations were carried out to confirm the presence of the compounds from the azaarene group in the fractions isolated from meat samples. Figure 4A presents the mass spectrum of B*a*Acr registered for the extract isolated from the meat sample. The spectrum from the Mainlib and NIST databases and the spectrum of the standard are also shown for comparison.

**Figure 5:**
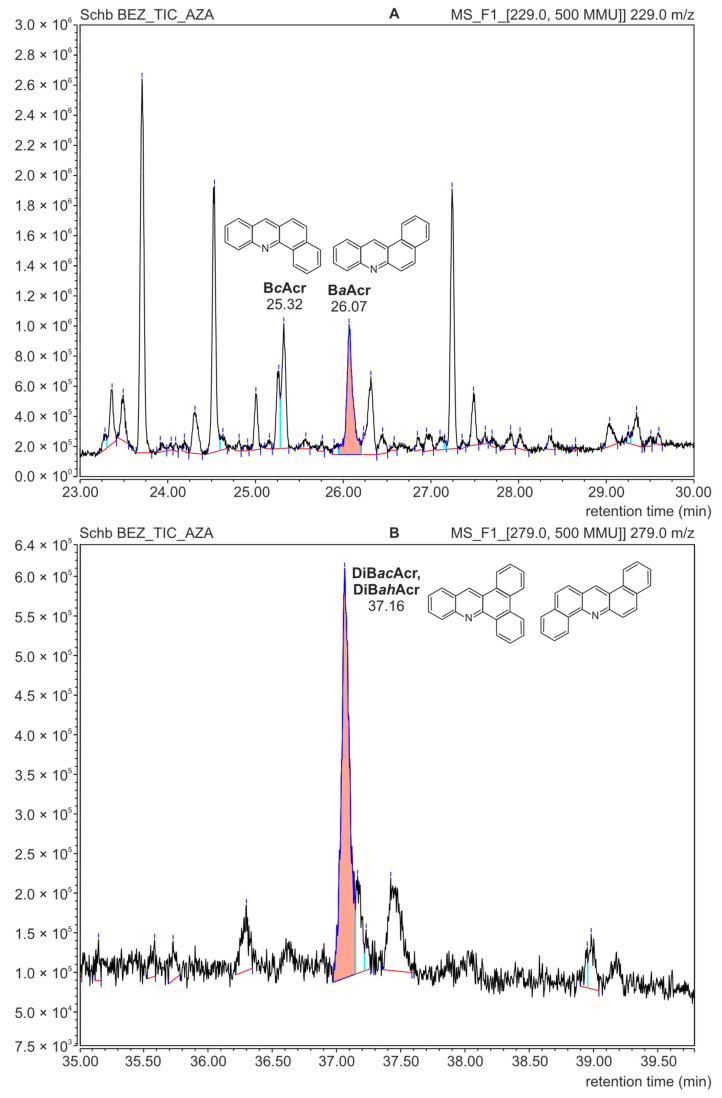
GC-MS mass chromatograms of the azaarene fraction separated from a roasted meat sample without additives; (**A**): recorded for the molecular mass of benzoacridines (m/z = 229); (**B**): recorded for the molecular mass of dibenzoacridines (m/z = 279).

The analysis using the GC-MS technique confirmed the presence of B*c*Acr and DiB*ac*Acr in the food samples. Figure 5 shows parts of the GC-MS chromatogram obtained for the fraction isolated from the meat sample without added fruit. Due to the low concentration of azaarenes, the record option “extract mass channel” was chosen from the total ion chromatogram. This allowed us to obtain mass chromatograms for m/z = 229 corresponding to the molecular mass of benzoacridines (Figure 5A) and m/z = 279 for dibenzoacridines (Figure 5B). High signals on these chromatograms were recorded at the same retention times as for the azaarene standards, i.e., 25.30 min (for B*c*Acr), 26.05 min (B*a*Acr) and 37.15 min (for the isomers DiB*ac*Acr and DiB*ah*Acr).

Although the concentrations of azaarenes in heat-treated meat are much lower than those of heterocyclic amines, efforts to reduce their concentrations in food are worthwhile. These compounds can be harmful to humans as the metabolites of some azaarenes, especially dibenzoacridines, are potentially carcinogenic in experimental animals [31,32].

It is worth noting that the application of plant components in high-protein food preparation can not only enhance the taste but, more importantly, make the dishes healthier. Fruits contain many valuable components: vitamins, polyphenol compounds, and mineral components. They have dietary fiber which can prevent alimentary tract neoplasm, and products of their metabolism show anti-bacterial and anti-inflammatory activity for the urinary system and alimentary system [66,78]. A diet rich in some fruits and vegetables may protect against the mutagenic activity of heterocyclic aromatic amines by means of the activation of P450 cytochrome enzymes in the process of detoxification [23,29,79]. The use of natural additives in the preparation of meat dishes can be a simple way to reduce the formation of carcinogenic compounds formed during the thermal processing of food and reduce human exposure to these compounds.

## 4. Conclusions

During the thermal processing of meat under common household conditions, carbolines (derivatives of pyridoimidazole and pyridoindole) and azaarenes (nitrogen heterocyclic derivatives of PAHs) are formed.

The analytical procedure of alkaline hydrolysis, extraction with diatomaceous earth, and solid-phase extraction using columns with a cation exchanger (PRS) and octadecylsilane (C_18_), allowed us to isolate the compounds that belong to these two groups, i.e., carbolines and azaarenes. Using high-performance liquid chromatography with fluorescence detection by applying excitation and emission wavelength programs which were selected for individual compounds, it was possible to determine them at a concentration level below 0.1 ng/g of meat.

Roasting the meat in an electrical oven using a roasting bag allowed us to obtain a dish in which the content of non-polar HAAs and azaarenes was small. The total concentration of the six determined carbolines and five azaarenes was 4.44 ng/g and 0.095 ng/g of meat, respectively.

The addition of dried fruit stuffing (prunes, dried apricots, and cranberries) to the pork loin reduced the content of δ-carbolines (Glu-P-1, Glu-P-2), γ-carbolines (Trp-P-1, Trp-P-2), α-carbolines (AαC, MeAαC), and azaarenes (benzoacridines and dibenzoacacridines). In contrast, the addition of dried fruit increased the concentration of norharmane and harmane.

## Figures and Tables

**Table 1:** Names and abbreviations of carbolines and azaarenes evaluated in the study.

Carboline	Abbreviation	Azaarene	Abbreviation
2-Amino-9H-pyrido [2,3-b]indole	AαC	Benzo[*a*]acridine	B*a*Acr
2-Amino-3-methyl-9H-pyrido [2,3-b]indole	MeAαC	Benzo[*c*]acridine	B*c*Acr
9H-pyrido [3,4-b] indole	Norharmane	Dibenzo[*a,c*]acridine	DiB*ac*Acr
1-methyl-9H-pyrido [3,4-b]indole	Harmane	Dibenzo[*a,h*]acridine	DiB*ah*Acr
3-Amino-1-methyl-5H-pyrido [4,3-b]indole	Trp-P-2	Dibenzo[*a,j*]acridine	DiB*aj*Acr
3-Amino-1,4-dimethyl-5H-pyrido [4,3-b]indole	Trp-P-1		
2-Aminodipyrido [1,2-a:3’,2’-d]imidazole	Glu-P-2		
2-Amino-6-methyldipyrido [1,2-a:3’,2’-d]imidazole	Glu-P-1		

**Table 2:** Concentrations (recovery corrected values) of β-carbolines in meat cooked with and without additives (ng/g). The results are presented as means ± standard deviations (SD), corresponding to duplicate HPLC analyses of fractions obtained by triplicate extractions (*n* = 6). Different letters in each column denote statistically significant differences (*p* < 0.05).

Pork Loin Sample	Norharmane	Harmane	Total Content
Without additives	2.16 ± 0.08 ^a^	1.00 ± 0.08 ^a^	3.16
With prunes	5.99 ± 0.23 ^b^	3.59 ± 0.09 ^b^	9.58
With apricots	7.42 ± 0.82 ^c^	1.83 ± 0.23 ^c^	9.25
With cranberries	12.32 ± 0.97 ^d^	1.20 ± 0.04 ^d^	13.52
Recovery (%) of spiked standards (RSD ^1^, %); *n* = 9			
Spiking level:			
10 ng/g	73.15 (8.2)		78.47 (12.8)
40 ng/g	74.36 (8.6)		84.28 (4.6)
Limit of quantification (ng/g of meat)			
	0.10		0.18

^1^ RSD—relative standard deviation.

**Table 3:** Concentrations (recovery corrected values) of δ-, α- and γ-carbolines in meat cooked with and without additives (ng/g). The results are presented as means ± standard deviations (SD), corresponding to duplicate HPLC analyses of fractions obtained by triplicate extractions (*n* = 6). Different letters (^a^, ^b^, ^c^, ^d^) in each column denote statistically significant differences (*p* < 0.05).

Pork LoinSample	Glu-P-2	Glu-P-1	AαC	MeAαC	Trp-P-1	Trp-P-2	Total Content (Inhibition %)
Without additives	0.27 ± 0.04 ^a^	0.31 ± 0.02 ^a^	0.16 ± 0.03	0.21 ± 0.03	0.18 ± 0.01 ^a^	0.15 ± 0.01 ^a^	1.28
With prunes	0.11 ± 0.02 ^b^	0.11 ± 0.02 ^b^	0.16 ± 0.03	n.q ^1^	0.03 ± 0.00 ^b^	0.02 ± 0.00 ^b^	0.43 (66.4)
With apricots	0.16 ± 0.02 ^c^	0.12 ± 0.02 ^b^	0.13 ± 0.03	n.q ^1^	0.09 ± 0.01 ^c^	0.04 ± 0.00 ^c^	0.54 (57.8)
With cranberries	0.16 ± 0.03 ^c^	0.19 ± 0.04 ^c^	0.12 ± 0.03	n.q ^1^	0.07 ± 0.01 ^c^	0.05 ± 0.01 ^d^	0.59 (53.9)
Recovery (%) of spiked standards (RSD ^2^, %); *n* = 9							
Spiking level:							
10 ng/g	45.49 (5.8)	43.46 (6.3)	48.06 (11.6)	43.76 (5.6)	45.24 (7.6)	58.0 (10.0)	
40 ng/g	46.04 (6.6)	44.25 (5.1)	47.67 (9.8)	43.72 (9.6)	47.93 (5.0)	60.26 (8.3)	
Limit of quantification (ng/g of meat)							
	0.1	0.05	0.05	0.10	0.03	0.01	

^1^ n.q—not quantified (below the LOQ). ^2^ RSD—relative standard deviation.

**Table 4:** Contents (recovery corrected values) of azaarenes in meat cooked with and without additives (ng/g). The results are presented as means ± standard deviations (SD), corresponding to triplicate HPLC analyses of fractions obtained by triplicate extractions (*n* = 6).

Pork Loin Sample	B*c*Acr + B*a*Acr ^1^	DiB*ac*Acr	DiB*ah*Acr + DiB*aj*Acr ^2^	Total Content (Inhibition %)
Without additives	0.068 ± 0.007	0.027 ± 0.003	n.q ^3^	0.095
With prunes	0.021 ± 0.003	0.023 ± 0.003	n.q ^3^	0.044 (53.68)
With apricots	0.066 ± 0.009	0.018 ± 0.002	n.q ^3^	0.084 (11.58)
With cranberries	n.q ^3^	n.q ^3^	n.q ^3^	n.q ^3^
Recovery (%) of spiked standards (RSD ^4^, %); *n* = 9				
Spiking level:	B*c*Acr	DiB*ac*Acr	DiBahAcr	
10 ng/g	65.87 (8.2)	65.69 (11.0)	66.83 (13.2)	
40 ng/g	71.93 (12.6)	69.46 (16.3)	66.77 (15.5)	
Limit of quantification (ng/g of meat)				
	0.01	0.01	0.006	

^1^ determined using a calibration graph recorded for B*c*Acr; ^2^ determined using a calibration graph recorded for DiB*ah*Acr; ^3^ n.q—not quantified (below the LOQ); ^4^ RSD—relative standard deviation.

## Data Availability

The data presented in this study are included in this article.
